# Supplementary material for: Attenuation of PKCδ enhances metabolic activity and promotes expansion of blood progenitors
Source: EMBO J. 2018 Nov 16;37(24):e100409. doi: 10.15252/embj.2018100409 (PMC6293338; doi:10.15252/embj.2018100409)
Supplement: Supplementary file 1 — Appendix [file EMBJ-37-e100409-s001.pdf]

Appendix

Table of Contents

Appendix Figure legends	page 2-4
Appendix Figure S1	page 5
Appendix Figure S2	page 6
Appendix Figure S3	page 7
Appendix Table 1	page 8
Appendix Table 2	page 9

Appendix Figure S1.

Increased frequency of bone marrow HSPCs in PKCδ-deficient mice.

A. Total BM cell numbers from two femurs and 2 tibias of *WT* or PKCδ KO mice. Data compiled from n=14 mice per each genotype.

B. Frequency of Lin-Sca1+ckit+ (LSK) cells, as a percent of live cells, in the fetal liver of embryonic day 14.5 (e14.5) embryos of mice of the indicated genotype (n=5 embryos analyzed per genotype). Data represent mean ± SEM., and indicate no statistically significant differences by one-way ANOVA analysis with subsequent Holm-Sidak's multiple comparison tests.

C. Quantification of colony formation from total BM cells harvested from *WT* (n=6) or PKCδ KO (n=6) mice. Colony forming unit cell (CFUc) frequencies were determined at day 12 after seeding in methylcellulose medium and presented as number per 20,000 BM cells. Each sample was plated in duplicate in each experiment. Data are pooled from 3 independent experiments and presented as mean ± SEM. $^{**}p<0.01$ by two-tailed Student's unpaired *t*-test analysis for comparison of control and PKCδ KO mice.

D. Schematic of the spleen colony forming unit assay (CFU-s). Bar graph represents the number of colonies formed per spleen at day 8 ($CFU\text{-}S_8$) and day13 ($CFU\text{-}S_{13}$) after transplantation of pooled total BM (n=2 donor mice for each genotype) into irradiated recipients (n=5 recipients for each genotype). Data presented as mean ± SEM. $^{**}p<0.01$ by two-tailed Student's unpaired *t*-test analysis for comparison of control and PKCδ cKO mice. As $CFU\text{-}S_8$ reads out primarily oligolineage progenitor cell activity, whereas $CFU\text{-}S_{13}$ reflects the presence of less differentiated multipotent progenitors and HSCs (Zhang et al., 2009), these data are consistent with a selective expansion of the most primitive HSPC subsets in PKCδ KO BM.

E,F Schematic of competitive transplantation assays using FACS-purified LT-HSCs (**E).** Percent of total donor-derived, hematopoietic cells ($CD45.2^+$), B-cells ($B220^+$), myeloid cells ($CD11b^+Gr1^+$), and T cells ($CD3^+$) in the peripheral blood (PB) of recipient mice, as determined by FACS at the

indicated time points (**F**). (n=5 recipients per genotype). No statistically significant differences in reconstitution were detected.

Data Information: All the data presented as presented as mean ± SEM. **p<0.01 by two-tailed Student's unpaired t-test analysis (**A-D**) or two-way ANOVA analysis with Sidak's multiple comparison tests (**F**).

Appendix Figure S2.

Augmented HSPC pool size and contribution to mature peripheral blood cells following loss of PKCδ in the hematopoietic system.

A. Experimental outline of competitive BM reconstitution assay.

B. Representative FACS plots show the percentage of total donor derived ($CD45.2^{+}$) cells in PB of recipient mice at 16-weeks post-pIpC treatment. Bar graph shows the percentages of total donor-derived cells in the PB of recipient mice at indicated times pre-and post-pIpC treatment. (n=7-8 mice per genotype).

Data information: Statistical significance was determined by repeated measures two-way ANOVA analysis with Sidak's multiple comparison tests for comparison of control and *PKCδ cKO* mice at each time point. *p<0.05 and **p<0.01.

Appendix Figure S3.

PKCδ sets a threshold for metabolic activation of HSPCs during myeloablative regeneration.

A. Experimental design.

B. Mitochondrial OCR rate in bone marrow HSPCs. MitoStress test revealed increased basal oxygen consumption rates (OCR) (measured before inhibitors treatment, left); the Maximal OCR capacity after FCCP treatment (middle), and the production of ATP (right) in LSKs from 5-FU treated $PKC\delta^{\Delta/\Delta}$ (cKO) mice as compared to LSKs from treated WT mice.

Data information: All data are presented as mean ± SEM (n=6 mice per genotype), *p<0.05 and

**$p<0.01$, by two-tailed Student's unpaired *t*-test analysis for comparison of control and PKCδ cKO mice.

Appendix Figure S1

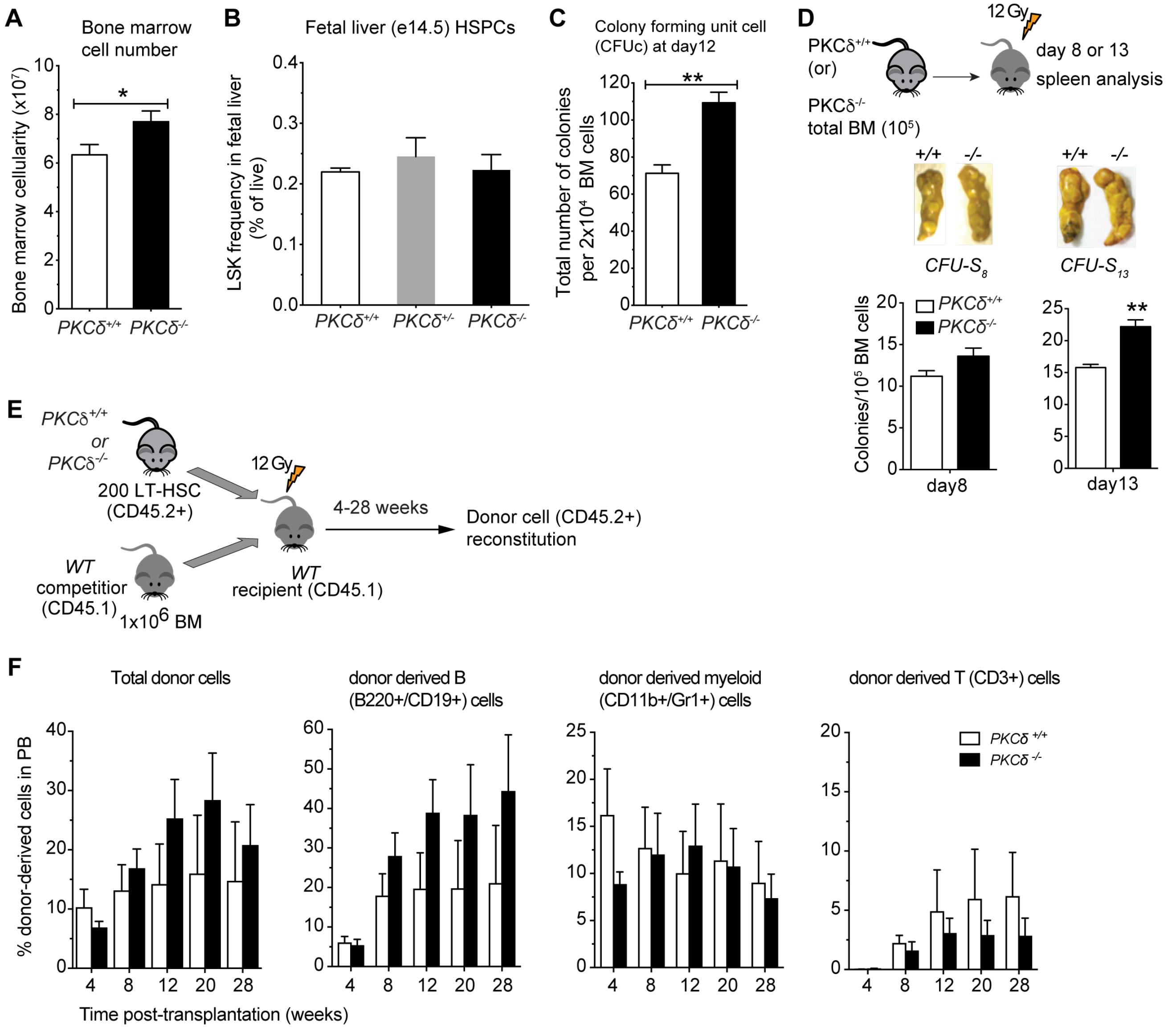

Appendix Figure S2

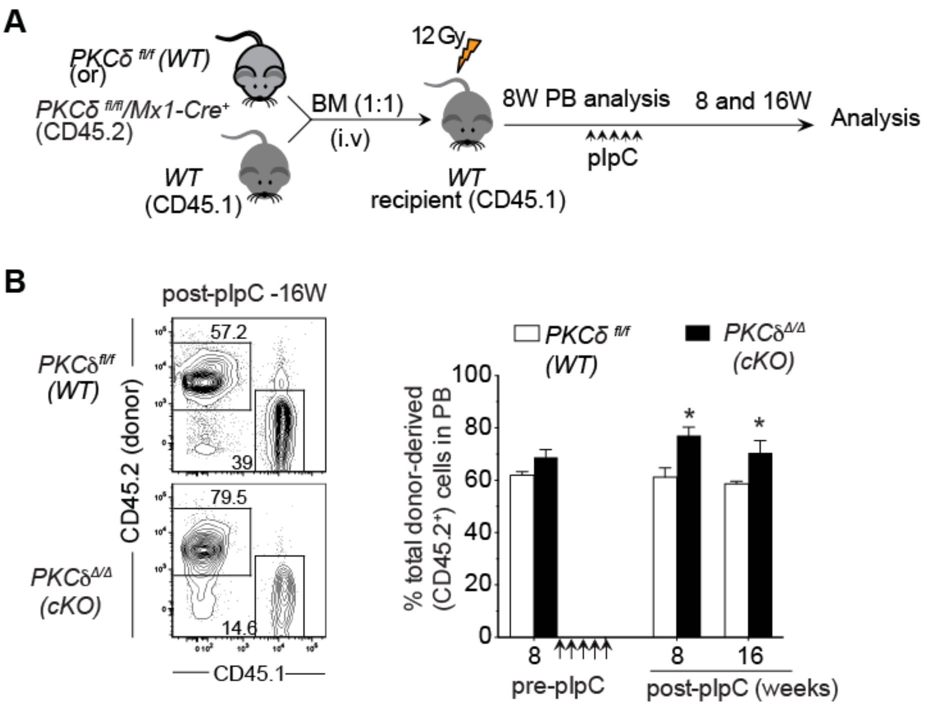

Appendix Figure S3

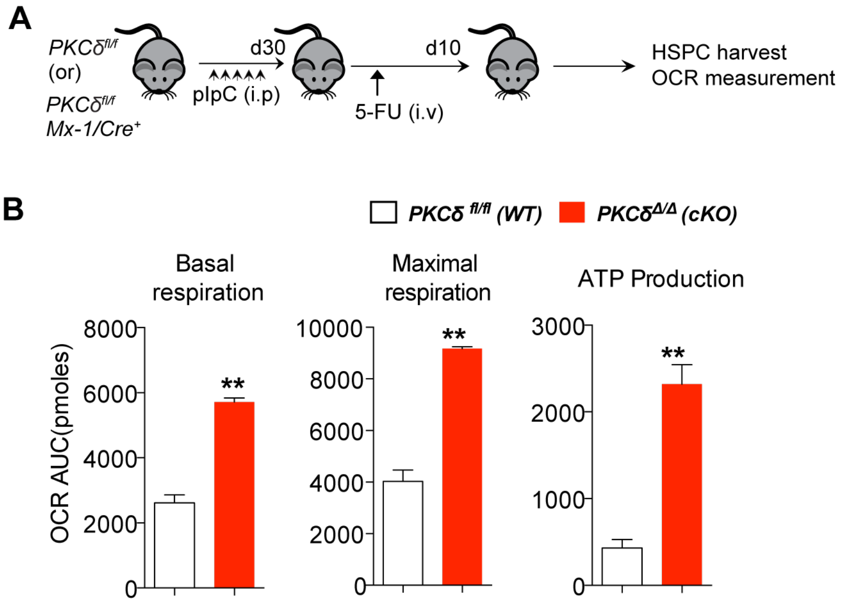

Appendix Table 1 (related to Materials and Methods and Figure 7). List of genes and primers used for RT-PCR analysis. All Taqman primers (FAM) were purchased from Applied Biosystems.

**Gene**	**Product ID**
Pdk2	Mm00446681_m1
Pdk4	Mm01166879_m1
Ldha1	Mm01612132_g1
Acads	Mm00431617_m1
Hes1	Mm01342805_m1
ATP5a1	Mm00431960_m1
Cox5a1	Mm01176957_m1
Cyc1	Mm00470540_m1
Cdkn2c	Mm00483243_m1
Cdkn1b	Mm00438168_m1
Mcl1	Mm00725832_s1
Bcl2l1	Mm00437783_m1
Egr1	Mm00656724_m1
Gfi1b	Mm00492318_m1
Bad	Mm00432042_m1
Cdkn2a	Mm00494449_m1
Cebpα	Mm00514283_s1
Nfe2L2 (Nrf2)	Mm00477784_m1
β*-Actin*	Mm00607939_s1
TBP	Mm00446971_m1
Gata1	Mm01352636_m1
Gata2	Mm00492301_m1
ID2	Mm00711781_m1
Pkm	Mm00834102_gH
Spi1 (PU.1)	Mm00488142_m1

Appendix Table 2. List of primary and secondary antibodies used for intracellular flow cytometry.

**Primary antibody**	**Supplier**	**Catalogue#**	**Dilution**
Phospho-Akt (Ser473) (D9E) XP® Rabbit mAb	Cell Signaling Technology	4060	1:200
PhosphoDetect™Anti-PDH-E1α (pSer293) Rabbit pAb	Millipore	AP1062	1:100
Phospho-FoxO3a (Ser253) Antibody	Cell Signaling Technology	9466	1:100
Phospho-S6 Ribosomal Protein (Ser235/236) (D57.2.2E) XP® Rabbit mAb	Cell Signaling Technology	4858P	1:100
Phospho-Rb (Ser807/811) (D20B12) XP® Rabbit mAb	Cell Signaling Technology	8516S	1:400
c-Myc(D84C12) Rabbit mAb (Alexa Fluor 647 conjugate)	Cell Signaling Technology	13871	1:50
Cyclin D1(92G2) Rabbit mAb	Cell Signaling Technology	2978	1:50
PPARγ (C26H12) Rabbit mAb	Cell Signaling Technology	2435	1:100
Anti-PPARδ (C26H12) Rabbit polyclonal Ab	Abcam	ab23673	1:100
Phospho-NF-κB p65 (Ser536) (93H1) Rabbit mAb (Alexa Fluor® 647 Conjugate) #488	Cell Signaling Technology	4887	1:50
Phospho-mTOR (Ser2448) (D9C2) XP® Rabbit mAb	Cell Signaling Technology	5536	1:50
Phospho-p38 MAPK (Thr180/Tyr182) (D3F9) XP® Rabbit mAb	Cell Signaling Technology	4511	1:400
Phospho-p44/42 MAPK (Erk1/2) (Thr202/Tyr204) (197G2) Rabbit mAb	Cell Signaling Technology	4377	1:200
Anti-rabbit IgG (H+L), F(ab')2 fragment (Alexa Fluor 647 conjugate)	Cell Signaling Technology	4414	1:500
Anti-rabbit IgG (H+L), F(ab')2 fragment (Alexa Fluor 488 conjugate)	Cell Signaling Technology	4412	1:500
