# Supplementary material for: Attenuation of PKCδ enhances metabolic activity and promotes expansion of blood progenitors
Source: EMBO J. 2018 Nov 16;37(24):e100409. doi: 10.15252/embj.2018100409 (PMC6293338; doi:10.15252/embj.2018100409)
Supplement: Supplementary file 2 — Expanded View Figures PDF [file EMBJ-37-e100409-s002.pdf]

Expanded View Figures

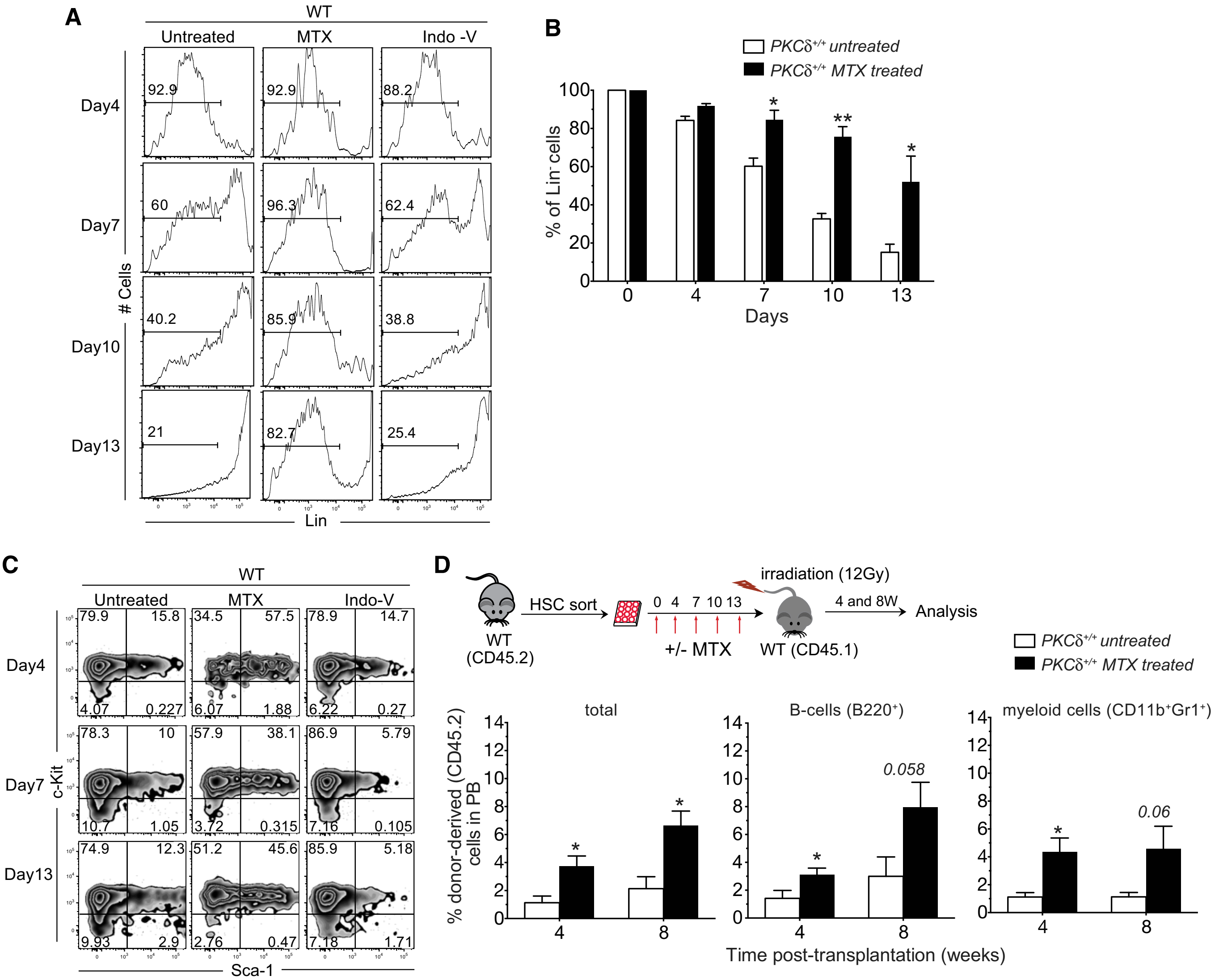

Figure EV1. Pharmacological modulation of PKCδ activity preserves HSPC activity *in vitro*.

A, B One hundred HSCs were sorted from WT mice and cultured in 96-well (U-bottom) plates in triplicate in the presence of mSCF (20 ng/ml) and mTPO (20 ng/ml) and with or without Mallotoxin (MTX, 5 μM) or Indolactam V (Indo-V, 10 μM) for indicated time. (A) FACS histograms and (B) bar graph show the percentage of Lin- cells (pre-gated on live cells).

C At the indicated time of culture, cells were analyzed for LSK phenotype. Representative FACS plots showing the percentage of cells retaining LSK phenotype. Data are representative of two independent experiments (*n* = 5 mice total per treatment group).

D Schematic of competitive reconstitution analysis of MTX-treated WT HSPCs. After 13 days in culture, cells were transplanted into recipient mice along with 1×10^6 competitive total BM cells ($CD45.1^+$). Percentage of total donor-derived cells ($CD45.2^+$), B cells ($B220^+$), and myeloid cells ($CD11b^+$ $Gr1^+$) in the peripheral blood was analyzed at indicated time (*n* = 6 mice per condition).

Data information: All data shown as mean ± SEM. *$P < 0.05$ and **$P < 0.01$ by repeated measures one-way ANOVA analysis with Bonferroni posttest.

© 2018 The Authors

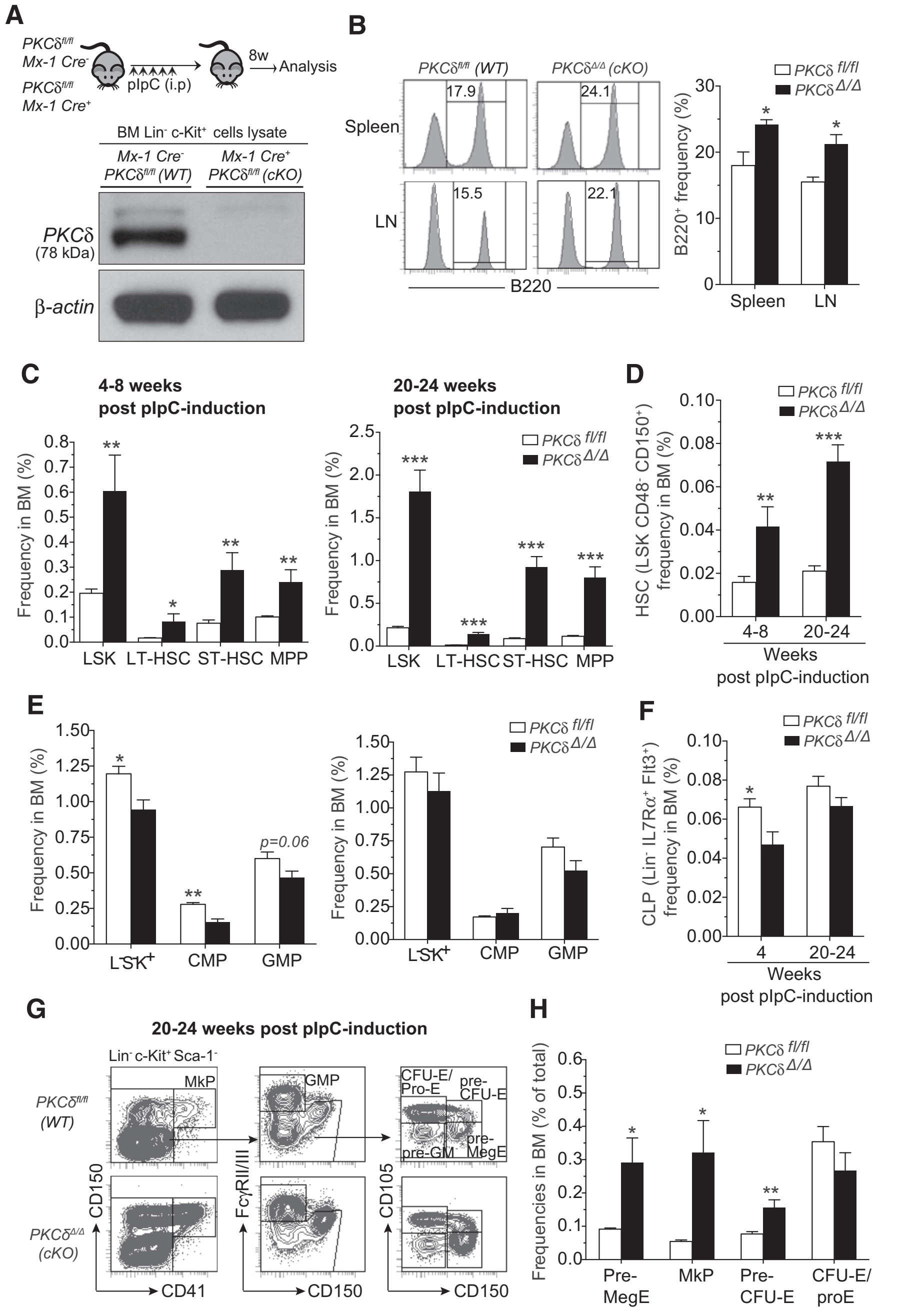

Figure EV2. Characterization of hematopoietic stem and progenitor cell subsets in the BM of WT and PKCδ cKO mice.

A Experimental design. Representative Western blot analysis for detecting *PKCδ* protein in $Lin^{-}Kit^{+}$ BM cells from indicated mice at 8-week post-pIpC treatment shows absence of *PKCδ* protein in cKO cells.

B FACS histograms show the frequency of $B220^{+}$ cells in spleen and lymph nodes of cKO mice at 24-week post-pIpC treatment (n = 6–8 mice per genotype).

C Increased frequency of HSPCs in the BM of *control* and *cKO* mice at 4–8 or 20–24 weeks after pIpC treatment (n = 8–9 mice per genotype and time point).

D, E Frequency of HSCs (HSC-SLAM) (D), and myeloid progenitor subsets (E) in the BM of *control* and *cKO* mice at 4–8 and 20–24 weeks after pIpC treatment (n = 8–9 mice per genotype and time point).

F Frequency of common lymphoid progenitors (CLPs) in the BM of *control* and *cKO* mice at 4–8 and 20–24 weeks after pIpC treatment (n = 6 mice per genotype and time point).

G Representative FACS plots show the gating strategy of MkP, Pre-MegE, MkP, Pre-CFU-E, and CFU-E/Pro-E subpopulations in the BM of *WT* and *cKO* mice at 24 weeks after pIpC treatment.

H Frequencies of indicated subsets in the total BM (n = 6 mice per genotype).

Data information: All data are presented as mean ± SEM, $*P < 0.05$, $***P < 0.01$, and $***P < 0.001$, by repeated measures two-way ANOVA analysis with Sidak's multiple comparison tests (B, D, and F) or by two-tailed Student's unpaired t-test analysis (C, E, and H).

© 2018 The Authors

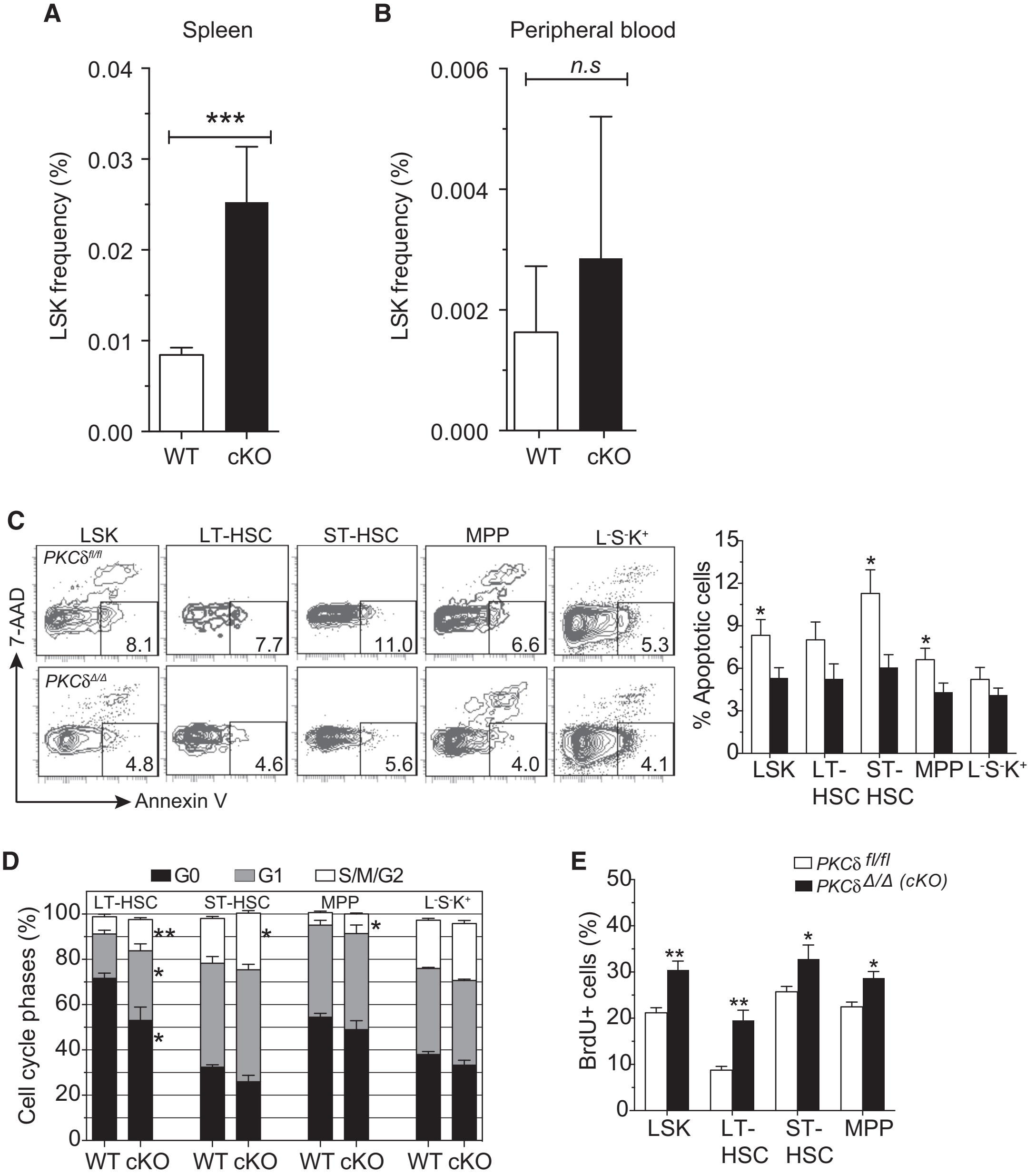

Figure EV3. Expanded HSPC pool size in PKCδ cKO mice.

A Frequency of LSK cells in the spleen of *control* and *cKO* mice at 20–24 weeks after pIpC treatment (*n* = 4–5 mice per genotype).

B Frequency of LSK cells in the peripheral blood of *control* and *cKO* mice at 20–24 weeks after pIpC treatment (*n* = 4–5 mice per genotype).

C Representative FACS plots and bar graph show the mean percentage of apoptotic cells in the indicated HSPC subsets of *PKCδ*$^{fl/fl}$ (control) and *PKCδ* cKO BM at 8 weeks after pIpC treatment (*n* = 7 mice per genotype).

D Cell cycle distribution of indicated HSPC subsets from *PKCδ*$^{fl/fl}$ (WT) and PKCδ cKO mice at 8 weeks after pIpC treatment (*n* = 7 mice per genotype) as assessed by Ki67-Hoechst staining.

E Short-term BrdU incorporation assay. Bar graph shows the percentage of BrdU$^+$ cells in each BM HSPC subsets of *PKCδ*$^{fl/fl}$ (control) and PKCδ cKO mice at 8 weeks after pIpC treatment (*n* = 7 mice per genotype).

Data information: All data are presented as mean ± SEM, *$P < 0.05$, ***$P < 0.01$, and ***$P < 0.001$, by two-tailed Student's unpaired *t*-test analysis.

© 2018 The Authors

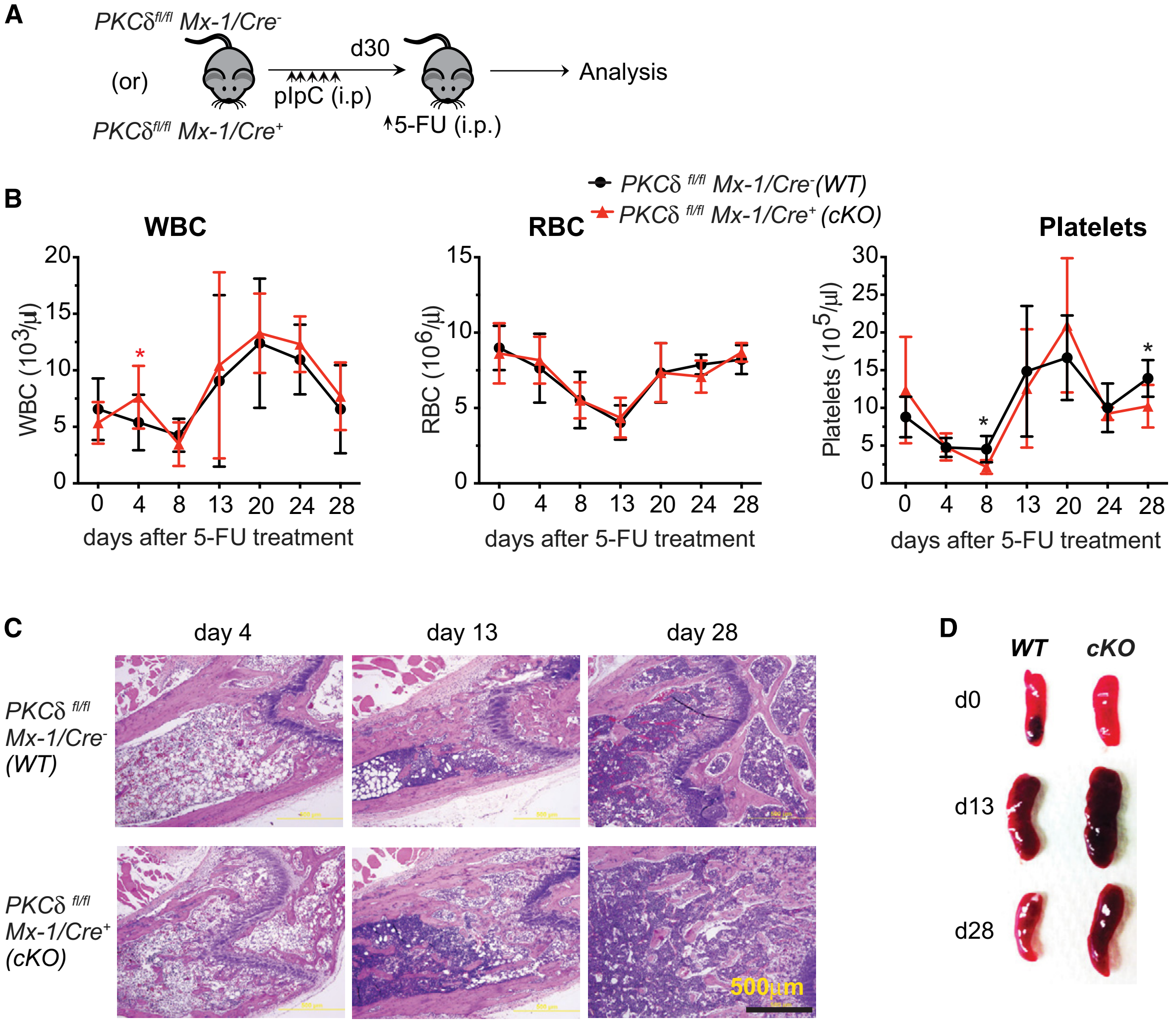

Figure EV4. Hematopoietic recovery in *PKCδ*$^{fl/fl}$ and *PKCδ* cKO mice after 5-FU treatment.

A, B (A) Experimental design, (B) kinetics of white blood cells (WBC), red blood cells (RBC), and platelet cell recovery in peripheral blood of WT and cKO mice after single dose of 5-FU treatment (n = 8–12 mice per genotype).

C Representative hematoxylin–eosin (H&E)-stained femur sections from indicated mice at day 4 of 13 and 28 after a single injection of 5-FU treatment (bottom) (n = 4–5 mice per time point for each genotype).

D Enlarged spleen in *PKCδ*$^{\Delta/\Delta}$ (cKO) mice during recovery phase (d13) after 5-FU treatment. Spleen size of WT and cKO mice at indicated time points after 5-FU treatment.

Data information: All data are presented as mean ± SEM, by repeated measures two-way ANOVA analysis with Sidak's multiple comparison tests (B) for comparison of control and *PKCδ cKO* mice at each time point. *$P < 0.05$.

© 2018 The Authors

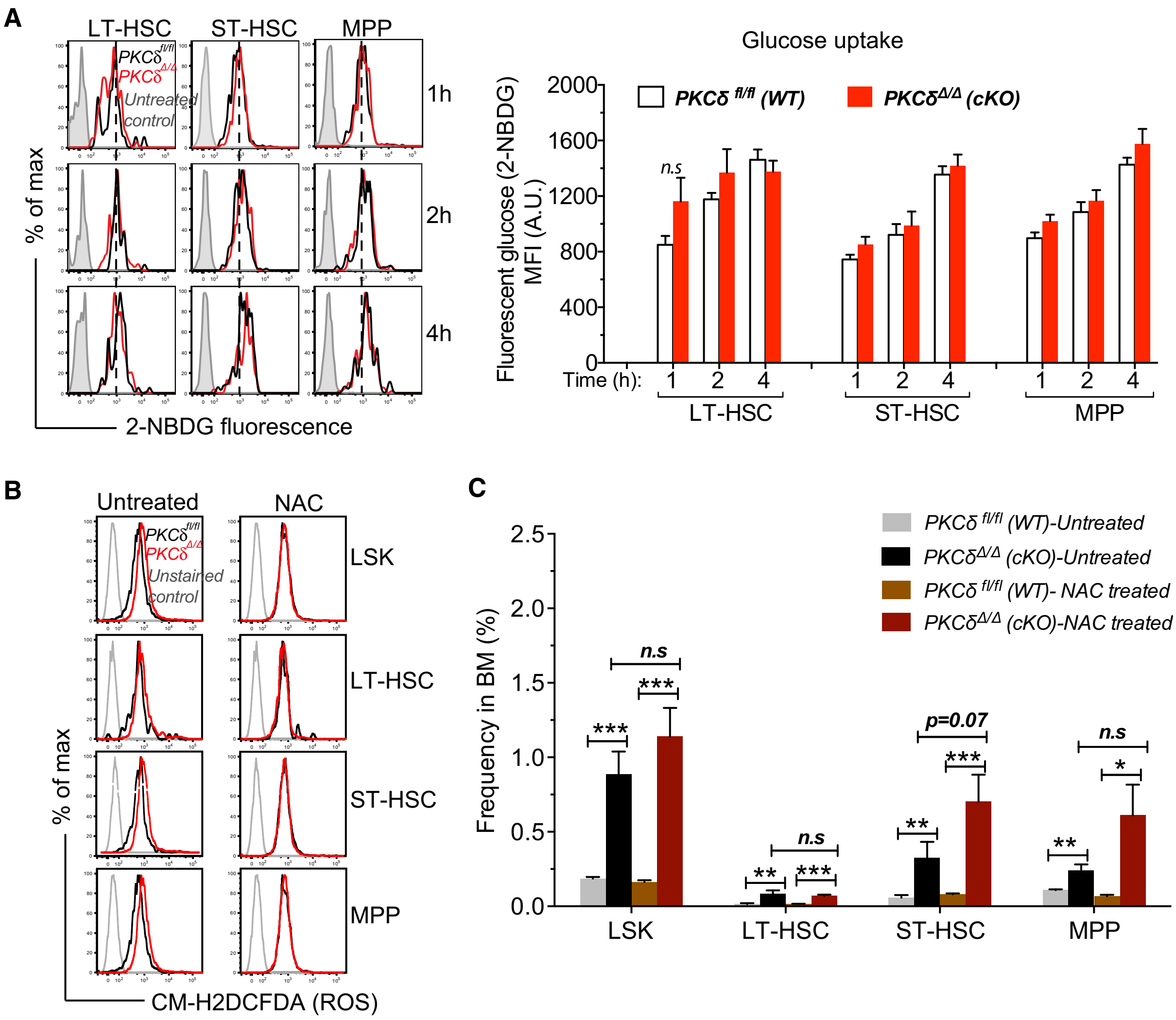

Figure EV5. Altered metabolic activity in PKCδ-deficient cKO HSPCs is not due to impaired glucose uptake, and inhibiting ROS levels via NAC treatment does not rescue increased HSPC pool size in PKCδ-deficient BM.

A FACS-sorted HSPC subsets were incubated with 2-NBDG for the indicated time. Representative histograms of 2-NBDG fluorescence at time indicated. Mean fluorescence intensity (MFI) of 2-NBDG fluorescence at indicated time. Cells treated without 2-NBDG used as controls. (n = 5 mice per genotype, mean ± SEM).

B NAC treatment rescued the increased ROS levels in PKCδ-deficient HSPCs. Representative histograms of CM-H2DCFDA fluorescence.

C NAC treatment did not rescue increased HSPC pool size in *PKCδ*-deficient BM. Bar graph represents HSPC frequency 8 weeks after NAC treatment. Untreated $PKC\delta^{fl/fl}$ and $PKC\delta^{\Delta/\Delta}$ (cKO) mice were used as controls (n = 6 mice per genotype).

Data information: All data are presented as mean ± SEM. Statistical significance was determined by repeated measures two-way ANOVA analysis with Sidak's multiple comparison tests (A and C). $*P < 0.05$ and $**P < 0.01$.

© 2018 The Authors
