# Supplementary material for: Attenuation of PKCδ enhances metabolic activity and promotes expansion of blood progenitors
Source: EMBO J. 2018 Nov 16;37(24):e100409. doi: 10.15252/embj.2018100409 (PMC6293338; doi:10.15252/embj.2018100409)
Supplement: Supplementary file 3 — Table EV1 [file EMBJ-37-e100409-s003.pdf]

Expanded view Table.1. Differential blood cell counts on peripheral blood from *PKCδ*$^{+/+}$ and *PKCδ*$^{-/-}$ littermates revealed no differences in hematologic parameters.

Parameter	*PKCδ*$^{+/+}$	*PKCδ*$^{-/-}$	*p*-value
WBCs (K/μL)	5.63±3.30	7.16±2.01	0.28
Neutrophils (K/µL)	1.40±0.91	1.97±0.74	0.19
Lymphocytes (K/μL)	3.91±2.21	4.78±1.60	0.39
Monocytes (K/μL)	0.26±0.19	0.30±0.13	0.68
Eosinophils (K/μL)	0.04±0.08	0.100±0.11	0.29
Basophils (K/μL)	0.01±0.02	0.02±0.03	0.31
RBC (M/μL)	9.69±0.67	9.73±0.89	0.92
Hb (g/dL)	14.64±0.77	14.36±0.99	0.52
HCT (%)	47.41±3.10	46.91±3.53	0.77
MCV (fL)	48.98±2.01	48.36±2.73	0.61
PLT (K/μL)	1,015.78±142	880.67±255.74	0.22

Values shown are means ±SEM (n=10 mice per group). WBC indicates white blood cell; RBC, red blood cell; Hb, hemoglobin; HCT hematocrit; MCV, mean corpuscular volume; PLT, platelets.
