# Supplementary material for: Attenuation of PKCδ enhances metabolic activity and promotes expansion of blood progenitors
Source: EMBO J. 2018 Nov 16;37(24):e100409. doi: 10.15252/embj.2018100409 (PMC6293338; doi:10.15252/embj.2018100409)
Supplement: Supplementary file 4 — Table EV2 [file EMBJ-37-e100409-s004.pdf]

Expanded view Table 2. Differential blood cell counts on peripheral blood from *PKCδ*$^{fl/fl}$ (control) and *PKCδ*$^{Δ/Δ}$ (*cKO*) mice after 8-20 weeks of pIpC induction revealed no differences in hematologic parameters.

**Parameter**	***PKCδ*$^{fl/fl}$ *(WT)***	***PKCδ*$^{Δ/Δ}$ *(cKO)***	***p*-value**
WBCs (K/μL)	6.56±2.6	7.35±1.7	0.21
Neutrophils (K/μL)	1.3±0.81	1.0±0.91	0.37
Lymphocytes (K/μL)	4.96±2.2	5.0±1.4	0.21
Monocytes (K/μL)	0.2±0.12	0.36±0.17	0.32
Eosinophils (K/μL)	0.06±0.1	0.1±0.15	0.46
Basophils (K/μL)	0.01±0.02	0.03±0.05	0.25
RBC (M/μL)	9±1.40	8.63±2.0	0.60
Hb (g/dL)	11.2±2.1	10.85±1.73	0.66
HCT (%)	43.5±6.45	42.4±7.2	0.72
MCV (fL)	48.43±2.24	49.7±3.5	0.26
PLT (K/μL)	772±293	1092±720	0.17

Values shown are means ±SEM. *PKCδ*$^{fl/fl}$ *(*control, n=12) and cKO (n=14 mice). WBC indicates white blood cell; RBC, red blood cell; Hb, hemoglobin; HCT hematocrit; MCV, mean corpuscular volume; PLT, platelets.
